# Study Protocol: A Cross-Sectional Examination of Socio-Demographic and Ecological Determinants of Nutrition and Disease Across Madagascar

**DOI:** 10.3389/fpubh.2020.00500

**Published:** 2020-09-17

**Authors:** Christopher D. Golden, Benjamin L. Rice, Hervet J. Randriamady, Arisoa Miadana Vonona, Jean Frederick Randrianasolo, Ambinintsoa Nirina Tafangy, Mamy Yves Andrianantenaina, Nicholas J. Arisco, Gauthier N. Emile, Faustin Lainandrasana, Robuste Fenoarison Faraniaina Mahonjolaza, Hermann Paratoaly Raelson, Vololoniaina Ravo Rakotoarilalao, Anjaharinony Andry Ny Aina Rakotomalala, Alex Dominique Rasamison, Rebaliha Mahery, M. Luciano Tantely, Romain Girod, Akshaya Annapragada, Amy Wesolowski, Amy Winter, Daniel L. Hartl, James Hazen, C. Jessica E. Metcalf

**Affiliations:** ^1^Department of Nutrition, Harvard TH Chan School of Public Health, Boston, MA, United States; ^2^Department of Environmental Health, Harvard TH Chan School of Public Health, Boston, MA, United States; ^3^Department of Global Health and Population, Harvard TH Chan School of Public Health, Boston, MA, United States; ^4^Madagascar Health and Environmental Research (MAHERY), Maroantsetra, Madagascar; ^5^Department of Ecology and Evolutionary Biology, Princeton University, Princeton, NJ, United States; ^6^Medical Entomology Unit, Institut Pasteur de Madagascar, Antananarivo, Madagascar; ^7^John A. Paulson School of Engineering and Applied Sciences, Harvard University, Cambridge, MA, United States; ^8^Department of Epidemiology, Johns Hopkins Bloomberg School of Public Health, Baltimore, MD, United States; ^9^Department of Organismic and Evolutionary Biology, Harvard University, Cambridge, MA, United States; ^10^Madagascar Country Program, Catholic Relief Services, Antananarivo, Madagascar; ^11^Princeton School of Public and International Affairs, Princeton University, Princeton, NJ, United States

**Keywords:** food security, micronutrient nutrition, planetary health, seasonality, migration, malaria, infectious disease, disease ecology

## Abstract

Madagascar has experienced significant environmental change since 1960, particularly through forest clearing for agricultural expansion. Climatic patterns are undergoing change in Madagascar as well, with increasing temperatures, droughts, and cyclonic activity. The impact of these environmental and climatic changes will pose threats to food availability, income generation, and local ecosystems, with significant potential effects on the spatial and temporal distribution of disease burden. This study seeks to describe the health status of a large sample of geographically and socially diverse Malagasy communities through multiple clinical measurements, detailed social surveys, and paired data on regional variation in local ecologies. With an increased understanding of the current patterns of variation in human health and nutrition, future studies will be better able to identify associations with climate and anticipate and mitigate the burdens expected from larger, longer-term changes. Our mixed-method approach included an observational cross-sectional study. Research subjects were men, women, and children from 1,125 households evenly distributed across 24 communities in four ecologically and socio-demographically distinct regions of Madagascar. For these 1,125 households, all persons of both sexes and all ages therein (for a total of 6,292 individuals) were recruited into the research study and a total of 5,882 individuals were enrolled. Through repeated social survey recalls and focus group meetings, we obtained social and demographic data, including broad categories of seasonal movements, and characterized the fluctuation of income generation, food production and dietary consumption. Through collection of clinical and biological samples for both point-of-care diagnoses and laboratory analyses, we obtained detailed occurrence (and importantly co-occurrence) data on micronutrient nutritional, infectious disease, and non-communicable disease status. Our research highlights the highly variable social, cultural, and environmental contexts of health conditions in Madagascar, and the tremendous inter-regional, inter-community, and intra-community variation in nutritional and disease status. More than 30% of the surveyed population was afflicted by anemia and 14% of the population had a current malaria infection. This type of rich metadata associated with a suite of biological samples and nutritional and disease outcome data should allow disentangling some of the underlying drivers of ill health across the changing landscapes of Madagascar.

## Introduction

Madagascar is characterized by high social, cultural, and ecological variation across its geography (1). This spatial variation is extreme, as rainfall varies 10-fold between regions of Madagascar, and some regions experience some of the highest levels of inter-annual variation in rainfall seen globally (2). There is also temporal variation, as seasonal cycles in food availability, wealth, human movement, and local ecologies drive shifting patterns in health through effects on nutrition, infectious diseases, and non-communicable diseases. Environmental and socio-demographic change will therefore alter the underlying determinants of nutritional status and infectious and non-communicable disease risk. The purpose of this study was to characterize variation in nutrition and disease risk across social and ecological settings in Madagascar.

Madagascar has experienced significant environmental change since 1960, particularly through forest clearing for agricultural expansion (3, 4). The pace of deforestation varies across time and geographies within Madagascar, and has been shown to increase during periods of political upheaval (5). The process of regular forest clearing and burning alters local ecologies, with downstream effects on water and soil. The adverse consequences of these ecosystem changes lead to challenges in conserving Madagascar's exceptional biodiversity [e.g., (6)], and they also significantly affect risks of malnutrition (7, 8) and disease (1, 9).

Climatic patterns are undergoing change in Madagascar as well. Historical records from 1961 to 2005 demonstrate an increasing temperature in 67% of locations across the island nation, with mean temperature expected to increase by 2.0–6.5°C by 2100 (10). Long-term trends indicate that temperature will increase, while rainfall is expected to increase in variability even further (1). Moreover, four general circulation models agree that the destructive potential of cyclones is expected to increase by 2–17% between 2060 and 2100 (10). Over the past 30 years, there have been more than thirty severe floods and five major droughts that have crippled the agricultural sector, killed hundreds of people and indirectly affected thousands to millions (1).

The impact of these environmental and climatic changes will pose threats to food availability, income generation, and local ecosystems, significantly affecting disease burden and the timing and magnitude of epidemics. Furthermore, the severity of impact will vary, in part, dependent on the baseline environmental, nutritional, and disease dynamics local to a region. However, few previous studies have characterized inter-regional variation in health in Madagascar. Therefore, this study seeks to describe the health status of a large sample of geographically and socially diverse Malagasy people through multiple clinical measurements and detailed social surveys. In addition, data were collected on regional variation in local ecologies, with a particular focus on mosquito vector habitats. Future studies of associations among climate, environment, and health can benefit from these new estimates and improve our ability to better anticipate and mitigate the burdens expected from larger, longer-term environmental and societal changes.

## Methods/Design

### Study Aims

In this study protocol we describe the methods used to (i) quantify variation in disease risk within and between rural communities in Madagascar, (ii) characterize potential socio-economic determinants of disease and nutrition status including food availability and human movement patterns, and (iii) estimate regional differences in the diversity and abundances of habitats used by mosquito vectors of disease. Previous studies of linkages between disease, nutrition, and environments in rural Madagascar, such as cohort studies in northeastern Madagascar (11, 12), were largely confined to a single ecological setting. This study aimed at collecting data across distinct ecological regions, using standardized methodology in a cross-sectional sample to allow the direct comparisons between regions. We first describe the study regions and the recruitment and enrollment procedures, then the methods relevant to collecting each data type, followed by a brief summary of the interim results.

Through focus group meetings (Supplementary Data Sheet 1) and social (Supplementary Data Sheets 2–4) and health (Supplementary Data Sheet 5) survey instruments, we obtained social and demographic data, including broad categories of seasonal movements, and characterized the fluctuation of income generation, food production and dietary consumption. Through collection of clinical and biological samples for both point-of-care diagnoses and laboratory analyses, we obtained detailed occurrence (and importantly co-occurrence) data on micronutrient nutritional, infectious disease, and non-communicable disease status.

Within the context of larger research programs, these data provide a much-needed first step toward future studies that will incorporate additional environmental variables (e.g., associating health outcomes with remotely sensed data including household proximity to forest and landscape composition).

Understanding the interconnections among environmental and climatic attributes, varying socio-cultural contexts, and the prevalence of malnutrition and disease is important to projecting future burdens, and planning interventions. The data collected over the course of this survey provide foundations to linking environmental and climatic data to nutritional and disease data in Madagascar, allowing development of integrated models of disease dynamics and their underlying risk factors.

### Study Design and Setting

#### Study Regions

**Figure 1:**
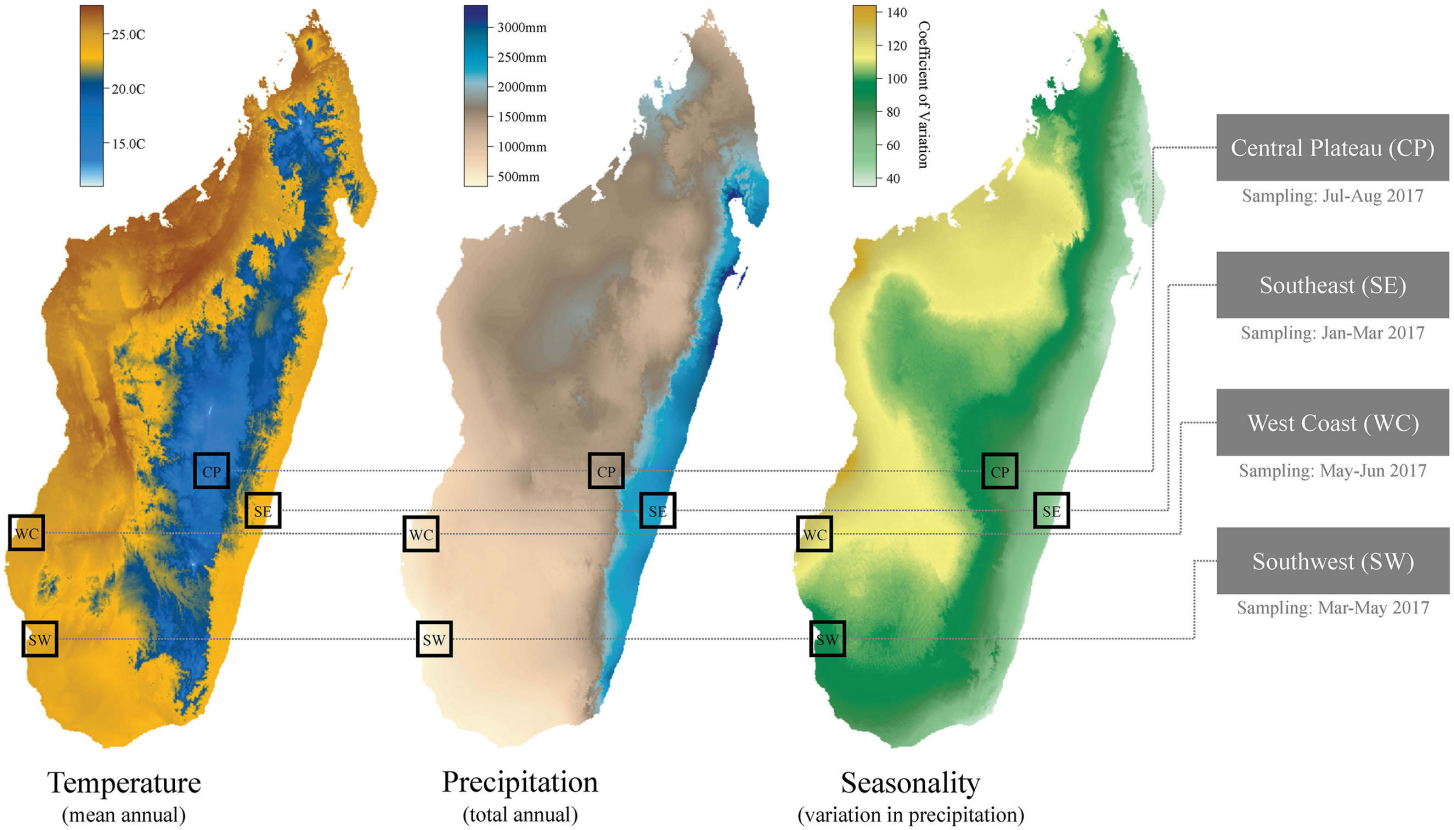
Climatic variation and regional focus for the cross-sectional study. Temperature and precipitation data sourced from WorldClim (14). Months of sampling listed below the four study regions.

Our mixed-method approach included an observational cross-sectional study. The regions sampled were selected to represent the major ecological regions within Madagascar. In Madagascar, gradients in elevation and varying patterns of precipitation (see Figure 1) create a hypervariable natural landscape across the 587,000 km^2^ island (2). The continuous variation in environmental variables across the county has been delimited into broad ecological regions with similar climatic and natural vegetation profiles. Generally, Madagascar can be divided into the higher elevation central plateau (wooded grassland-bushland mosaic and terraced agriculture), the high rainfall east coast (humid rainforest, secondary forest and flooded lowland rice paddy agriculture), the seasonally dry west coast (dry deciduous forest and secondary grasslands), and the more arid south and southwest (dry spiny forest-thicket and secondary grasslands) (11, 12). These ecological regions align with those used by government departments to separate the country into areas for aggregating health data or interventions [for example, the national Malaria Indicator Surveys; (13)].

Research subjects were men, women, and their children from 1,125 households evenly distributed across 24 communities in four ecologically and socio-demographically distinct regions of Madagascar (the southeast, southwest, west coast, and central plateau) (see Figure 1). These communities had already been enrolled in USAID-funded Catholic Relief Services (CRS) and Adventist Development and Relief Agency (ADRA) interventions aimed at reducing food insecurity through multiple pathways. CRS interventions include community-based nutrition and health activities, agriculture and livelihoods support, natural resource management, and disaster risk reduction and response activities. CRS and ADRA worked in every community in selected districts. Regional priorities were selected by the USAID mission as areas of food security challenges. Our team then used a stratified approach to randomizing communities in selected districts, after classifying communities by their proximity to the nearest town (greater than or less than 20 km) with an equal number of sites in each study region in each category (within 20 km of the city or more than 20 km from the city). Particular environmental features (e.g., forests, protected areas, rivers, etc.) were not used in selecting communities. Variation in community population size was not recorded but all communities were small, rural, agricultural settlements (*tanana*) that are typical of these areas and had ~50 to 250 households.

We refer to the four regions sampled in this study by their geography (see Table 1 for their corresponding administrative regions, sample sizes, and climatic variables): (i) the southeast (SE), (ii) the southwest (SW), (iii) the west coast (WC), and (iv) the central plateau (CP). Note that the southwest and west coast regions we distinguish in our study are located within the same, large, administrative region of Madagascar (Atsimo Andrefana), but we consider them as separate ecological regions in this study due to their geographic distance (~200 km) and marked differences in climate and vegetation. For example, total annual rainfall in the WC is approximately double that of the SW (Table 1), due to a large seasonal swing in rainfall in the WC (Figure 1).

**Table 1:** Study regions for the cross-sectional study in rural Madagascar.

**Region**	**Administrative region^a^**	**District(s)**	**Lat^b^**	**Lon^b^**	**n^c^ HH**	**n^c^ IND**	**Mean temp^d^ Min-Max**	**Annual rainfall^d^**
SE	Vatovavy Fitovinany	Mananjary	−21.26	48.24	309	1,665	19.0–27.3C	2,427 mm
SW	Atsimo Andrefana	Toliara	−23.03	43.79	294	1,602	18.1–30.4C	344 mm
WC	Atsimo Andrefana	Morombe	−21.74	43.55	281	1,461	18.2–31.5C	649 mm
CP	Amoron'i Mania	Ambatofinandrahana, Fandriana, Ambositra	−20.51	47.21	241	1,564	11.5-−22.9C	1,489 mm

a *Government defined administrative divisions (faritra)*.

b *Latitude and longitude of the approximate center of the study region*.

c *Number of households (HH), and individuals (IND) recruited in each study region*.

d *Mean annual minimum and maximum temperature (C) and total annual rainfall (mm) (14)*.

#### Recruitment and Enrollment

Within the 24 sampled communities, we randomly sampled ~50 households in each community for a total of 200–300 households per region, and a total household enrollment of 1,125 across all regions. For these 1,125 households, all persons of both sexes and all ages therein (for a total of 6,292 individuals) were recruited into the research study and 5,882 individuals were enrolled. There was no screening based on race or ethnicity. Households were defined as regularly cohabitating groups of individuals that included at least one reproductive aged woman and a child. This definition of households was used as it aligns with the typical household groupings in these areas and reproductive aged women (women 13–45 years of age) and young children (children 5 years of age and younger) are most sensitive to malnutrition (15, 16) and certain types of infectious and communicable disease risk [e.g, malaria; (17)].

Subjects were offered no compensation for participating in interviews or providing clinical samples at the primary sampling time, but were offered 1,000 Malagasy ariary (~$0.28USD) at the follow-up interview performed the following year. This amount of money was compensation for time lost from labor activities due to participation in the follow-up survey and was deemed to not be coercive.

We recruited individuals with a two-stage opt-out procedure; individuals could opt-out prior to enrollment that preceded the questionnaire portion of the study or prior to the subsequent biological sampling portion of the study. Initially, one or more local authorities, typically local community leaders, chiefs (*president-fokontany*), or community elders, accompanied the field research manager (BLR) to conduct a community meeting where speeches were given to describe the work. This was the only culturally appropriate way to describe our research and allow for questions and answers to be heard by all community members. Following this meeting, we used the community census provided by the local authority to randomize households for participation. Households without reproductive aged women and/or children under 5 years of age were excluded. Randomization occurred by assigning numbers to households in the community and then using a random number generator to select households to enroll. In some cases, community censuses were out of date or inaccurate. For these cases, community censuses were updated to include all households by conferring with community leaders and heads of local family groups.

Selected households were then visited by the investigator and the local community authority to invite participation. After a verbal summary of the study was presented by a member of the study team, individuals within selected households expressing interest in participation were invited to review a more detailed explanation of the study. For adults that were not literate, verbal explanations were provided. Informed consent was obtained from adults, verbal assent was obtained from children over 12 years of age, and permission was obtained from parents or guardians of younger children (see below for ethical approval declarations). Interviews and clinical measures were performed in private and lasted no more than 1–2 hours in total. The local authority was not present during the interviews or clinical assessments. Not showing up for the interview was viewed by our team as the subject declining to be interviewed.

In addition to household surveys, our team also conducted community-level focus groups where we asked 4–6 adults of each gender to participate in providing some basic information about the community. These focus groups covered topics such as distance to infrastructure (roads, hospitals, etc.), general comments on local practices relevant to nutrition and disease (how people cope with food insecurity, etc.), and characterizing local markets (determining prices and access to different goods, etc.).

Our team also conducted biological sampling. A blood sample was obtained from all enrolled individuals who were willing to provide a blood sample. Not showing up for the blood sampling portion of the study was viewed by our team as the subject declining to participate in this portion of the study. Individuals were sampled in the early morning, prior to their first meal, so that micronutrient concentrations in plasma would not be affected by recent eating and to not disrupt individuals' labor during the day. Blood samples were used for point-of-care malaria diagnoses and hemoglobin testing for anemia, as well as plasma collection and dried blood spot preservation for other analyses of disease and nutritional markers (Table 2). Individuals testing positive for malaria by point-of-care rapid diagnostic test (RDT) or hemoglobin testing results indicative of anemia were offered treatment and a consultation with a physician. Individuals deemed by physicians to require additional treatment after point-of-care testing were referred to a hospital.

**Table 2:** Criteria and metadata of all surveys and assessments.

**Surveys**	**Details**	**Age group targeted**
Dietary Intake	24-h and 1 week food recalls	All
Food Security	Coping strategies index	Head of household
	Household Food Insecurity Access Scale	
	Food availablity and market access	
Socio-economic status	Monthly recall of agricultural and cash crop sales, wages, and other sources of income	Head of household
Health related risk factors and behaviors	Mosquito bednet usage	All
	Proximity to healthcare infrastructure and care seeking behavior	All
	Access to water and sanitation	All
	Contact with wildlife and zoonotic disease exposure	All
	Pregnancy and breastfeeding	Reproductive aged women
***Clinical assessment***		
Anthropometry	Height/length	All
	Weight	All
	Mid-upper arm circumference	Children 5 and under
	Cranial circumference	Children 2 and under
Blood pressure	OMRON 10 Series monitor	Adults 16 and older
Temperature	Infrared thermometers	All
Malaria	Rapid diagnostic tests from SD Bioline Malaria	All
	Dried blood spots for molecular analysis	
Anemia	HemoCue201+	All
Nutritional biomarkers	Fatty acid profiles from OmegaQuant	All
	Ferritin	
	Transferrin receptor	
	Zinc	
	Retinol	
	Vitamin B12	
	Folate	
Inflammation biomarkers	Alpha glycolic protein	All
	C-reactive protein	
Intestinal parasites	Fecal sample microscopy	All

Blood samples were obtained through venous blood draw by trained, Malagasy medical staff (details on blood sample processing methodology are discussed below). However, we did not collect venous blood draws from the following: children under 2 years of age, whom the physician deemed too small for a blood draw and men and women 55 years of age and older. Only finger pricks were collected from these groups. Additionally, for individuals where the venous blood draw failed to obtain a sample, individuals wanting to receive point-of-care diagnoses were offered the choice to provide a finger prick sample.

Primary sampling took place between January and August 2017. Questionnaires, biological sampling, and focus groups were conducted in unison for a community such that all data collection for all households (and the individuals therein) in a community was completed over a maximum interval of seven days. Communities were sampled consecutively such that all communities in a region were sampled within a 6-week interval. Follow-up questionnaires were performed in each region from January to December 2018, every 2–3 months, in order to estimate intra-annual variation.

#### Household Dietary Intake

To understand the importance of dietary intake to human nutritional outcomes, we utilized a mixed-methods approach to quantify both household and individual levels of consumption (Table 2). Heads of household were asked to complete an adapted version of the Coping Strategies Index and the FAO's Household Food Insecurity Access Scale (18). Information was also gathered on crops grown and sold, and from a focused module on natural resource extraction including fishing and hunting. Dietary recalls (both 24 h and 1 week) were completed for every individual enrolled in the study at the time of the clinical sample, and then three or more additional times at 2 to 3-month intervals following the initial survey to capture seasonal differences in food availability and consumption [following protocols of (19)].

#### Clinical and Anthropometric Measures

The members of each household were invited to participate in the clinical and diagnostic aspects of the health research. A local healthcare professional recorded the following anthropometric measurements for each child: height/length, weight, and mid-upper arm circumference for children 5 years of age and under, and cranial circumference for children 2 years of age and under (Table 2). Height and weight were also recorded for adult women and men. Blood pressure was measured for all individuals over 16 years of age using an OMRON 10 Series (Model BP786N) monitor and temperatures were recorded using infra-red thermometers for all subjects. Subjects traveled (always less than a 30-min walk) to a private room where the health assessments were conducted. Lidocaine was applied to the arm's surface to dull the pain of needle insertion when individuals feared the pain of the blood draw. Venous blood draws were collected in one Sarstedt S-Monovette tube (5–7 mL) with lithium heparin and then processed using previously established protocols (19, 20). This whole blood was used to source blood for: (i) rapid diagnostic tests (RDTs) for malaria (a fingerprick was used if no venous blood draw was taken) (SD Bioline Malaria Ag P.f/Pan RDT), (ii) hemoglobin status through the use of a HemoCue 201+, (iii) a thin blood smear for parasitemia counts for malaria and cell counts (iv) dried blood spots on Whatman filter paper FTA cards (two spots per individual), and (v) one drop on OmegaQuant filter paper treated with HUFASave^TM^. The remaining whole blood was then separated by centrifuge into plasma and a blood pellet, with the plasma being aliquoted into 1 or 2 (depending on quantity of plasma obtained) 1.8 mL cryotubes that were then preserved in liquid nitrogen prior to freezing at −80C.

#### Nutritional Biomarkers

One aliquot of frozen plasma was shipped to the Western Human Nutrition Research Center (USDA) for nutritional analyses while the remaining plasma was stored at the Harvard T.H. Chan School of Public Health for future disease (e.g., serology for viral disease) tests. For nutritional analyses we analyzed for content of ferritin, transferrin receptor, zinc, vitamin A (retinol), folate, and vitamin B12 and will continue to be analyzed for other potential nutritional targets. Inflammation markers such as alpha glycolic protein (AGP) and C-reactive protein (CRP) were also measured here to control for the role of inflammation in affecting nutritional status. Dried blood from OmegaQuant filter paper was used to characterize fatty acid profiles for each sampled individual following established protocols (21). We were unable to prospectively conduct power calculations for nutritional outcomes for our study population because we did not have any baseline estimates of deficiency.

#### Disease Analysis

Processing of dried blood spots on Whatman FTA cards involved the extraction of nucleic acid material from the dried blood spot and genetic analysis will be performed to determine the presence or absence, as well as genotypes, of *Plasmodium* malaria parasites. *Plasmodium* genotype data will be used to characterize malaria population genetic diversity to infer estimates of transmission histories of communities. For stratification purposes only, genotyping of specific human loci known to impact the likelihood of malaria infection [e.g. hemoglobin type; (22)], Duffy-binding protein detection (23), etc. will be performed.

In addition, we collected a fecal sample from individuals enrolled in the study by providing fecal sample tubes (with spoons) and allowing individuals to collect this themselves by defecating and then spooning a sample into a tube. Approximately 83% of individuals returned a fecal sample. These fecal samples were stored in 90% ethanol and shipped to the Harvard T.H. Chan School of Public Health for microscopic analysis for intestinal parasite detection.

Plasma samples stored in cryotubes at −80C will be tested for antibodies to viral pathogens in order to evaluate the force of infection associated with a set of target pathogens, including interactions between nutritional and immune status. This will allow us to characterize the landscape of immunity for these focal pathogens [sensu (24)].

#### Questionnaire Response Data

For each enrolled household, one surveyor from the four surveyors on the research team first asked the head of household basic demographic questions about all members of the household (Supplementary Data Sheet 2). From this, a household census was created and each individual in the household was assigned an anonymous, unique individual ID code. All participants in a household were then asked to respond to a series of demographic, socio-economic, health, and nutrition questions (Supplementary Data Sheets 2–5). Additional subsets of questions relevant to only specific members of the household were asked, including questions particular to heads of households, all adults, or reproductive aged women (e.g., questions about pregnancy and breastfeeding). Prior to participation in the blood draw portion of the study, all participants were asked a set of questions about current health status, medication history, vaccination history, and disease risk associated behaviors (e.g., mosquito bednet usage). For younger children, typically under the age of nine, surrogate responses from a parent or responsible family member were used.

Interview length depended on the age and sex of the individual participant, with interviews of heads of households being the longest (~30–45 min in total) due to the inclusion of questions regarding general household characteristics in addition to the base set of questions asked to all individuals. Interviews of children were briefer, averaging ~10–15 min in total.

Supplementary Data Sheets contain the full set of questions asked to heads of households, adults, and all individuals (English translations of the Malagasy question prompts are shown). Questionnaires were administered verbally, in local dialects in Malagasy, and responses were recorded on a tablet (Samsung Galaxy Tab A) using KoBoCollect software. Surveyors were trained to adapt the text of questions to local dialects, though in some cases French language words were the most commonly used locally and so the question prompts occasionally contain a mixture of Malagasy and French wording.

#### Local Ecologies: Vector Habitat Mapping and Larval Sampling

To investigate the ecology of disease vectors, our team (led by NJA) characterized the peri-domicile larval habitat of mosquitoes in each of the 24 study communities using two methods: (1) a grid-based system to search for sources of mosquito vector larvae within 25 m of households; and (2) stratified transects. In sites where households were tightly clustered, we conducted a systematic habitat search of the clustered area and 25 m beyond the perimeter of households on the edges of the cluster. In sites with less concentrated households, we conducted habitat searches in a circular area of radius 25 m around each household. All mosquito larval habitats were mapped and geocoded using a Garmin Oregon 550t. In addition to mapping the presence/absence of larval habitats, we also conducted transect surveys to identify the larval habitats and species composition of anopheline mosquitoes endemic to each local ecology of each research site. Two 100 m transects were mapped within one undisturbed area and one area that represented the dominant human-altered land-use type in each study community. All mosquito larval habitats and distinct changes in land-use along the transect and 5 m to each side of the transect line were geocoded.

In both protocols, all habitats containing water were sampled for larvae using dippers and pipettes in a standardized method (1). Larvae were collected and stored in 95% ethanol. Information on the presence of water, the presence of other animals, the habitat's dimensions, the type of habitat, and the presence and number of eggs and pupae at the time of sampling was recorded. Additionally, all early and late instar larvae were enumerated. All larvae were sorted by genus and instar prior to identification. All 3rd and 4th instar *Anopheles* larvae were identified morphologically to the lowest possible taxonomic level at the Institut Pasteur de Madagascar (led by MLT). These vector data were combined with ecological and environmental data to characterize the distribution of vectors of infectious disease in these regions.

## Interim Results

**Figure 2:**
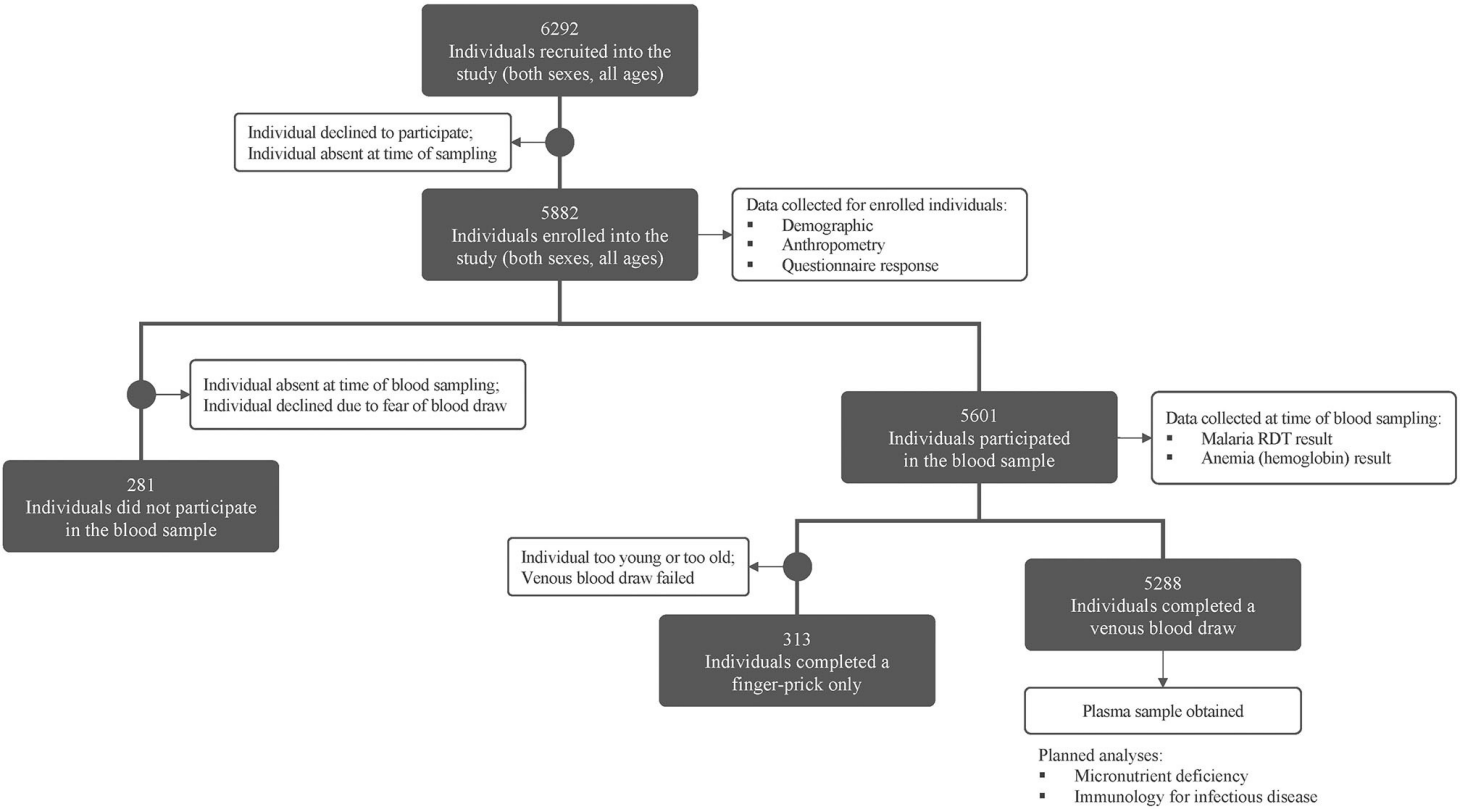
Consort figure for study enrollment and sampling. Enrollment and blood sampling in the MAHERY cross-sectional study. See methods for a full description of all sample types and data collected.

A total of 6,292 individuals (both sexes, all ages) within 1,125 households were recruited in the study (see Figure 2). Demographics and preliminary anemia and malaria data are shown in Table 3. Of the 6,292 enrolled individuals, 5,601 individuals (89.0% of the total enrollees) also consented/assented to and were present to participate in the blood draw portion of the study.

**Table 3:** Demographic and disease status summary statistics of the enrolled study population.

**Category**	**Value**	**SE^g^**	**SW**	**WC**	**CP**	**All**
Demographic information	*n*	1,601	1,493	1,384	1,404	5,882
Age	Mean (years)	19.1	16.6	16.6	18.1	17.6
	SD (years)	17.1	15.5	15.7	16.8	16.3
Sex	Female (%)	56.0	53.4	52.7	53.5	53.9
Age of first marriage for women	*n*^a^	243	257	233	284	1,017
	Mean (years)	19.6	17.1	17.3	19.1	18.3
	SD (years)	3.3	2.2	3.2	3.0	3.1
Age of woman's spouse at first marriage	*n*^b^	159	145	133	187	624
	Mean (years)	24.2	22.7	22.1	23.6	23.2
	SD (years)	4.6	5.1	5.2	4.7	4.9
Household head's monthly income	*n*^c^	305	294	281	241	1,121
	Mean (USD)	$35.23	$44.03	$28.69	$65.77	$42.46
	Median (USD)	$11.27	$22.53	$9.86	$14.65	$14.08
Anemia	*n*^d^	1,441	1,386	1,267	1,334	5,428
Anemia (mild, moderate, or severe combined)	%	45.1	31.6	33.5	9.4	30.2
Mild	%	22.1	17.6	17.4	6.1	15.9
Moderate	%	19.8	13.1	15.4	3.4	13.0
Severe	%	3.2	0.9	0.8	0.0	1.3
Malaria (by RDT*)*	*n*^e^	1,470	1,409	1,306	1,367	5,552
Positive	%	21.7	5.3	29.4	0.4	14.1
Reproductive aged women	*n*^f^	168	373	324	383	1,248
Pregnant	%	11.3	7.5	5.6	7.1	7.4
Breastfeeding	%	23.8	26.8	38.9	32.4	31.3

a *Women who recalled their age at the time of their first marriage*.

b *Women who recalled both their age and the age of their spouse at the time of their first marriage*.

c *USD conversion from Malagasy ariary using “Banky Foiben'i Madagasikara” (Central Bank of Madagascar) daily average exchange rate in 2017 (25)*.

d *Individuals over 6 months of age with a hemoglobin measurement, categorized using the standard WHO thresholds for age and sex groups (26)*.

e *Individuals with a valid rapid diagnostic test (RDT) result*.

f *Among women reporting that their first menstruation event had previously occurred*.

g *SE, southeast; SW, southwest; WC, west coast; CP, central plateau*.

The most common reasons individuals did not participate in the blood draw portion of the study were: (i) absence from the community during the time blood draws were performed, often due to fishing, hunting, or other resource-extraction activities; or (ii) declining participation due to a fear of blood draws; or (iii) cultural taboos. Among all enrolled individuals, 52.8% were female, however females contributed only 35.6% of the individuals who did not perform a blood draw. Of the males who missed the blood draw, a vast majority (71.0%) were adults, indicating that men were more likely to be absent from or decline participation in the blood draw.

Of the 5,601 individuals who participated in the blood draw portion of the study, a venous blood draw was performed for 5,288 individuals (94.4%) while a finger prick blood sample only was obtained for the remaining individuals (due to their being too young, too old, or due to failed venous blood draws, see above for blood draw methodology).

## Discussion

This study aimed to collect data on the distribution of nutritional and disease outcomes among men, women, and children of all ages in rural communities across varying ecological settings in Madagascar. Successful recruitment, enrollment, and sampling of between 1,308 and 1,482 individuals per region for four ecologically distinct regions of Madagascar provides an opportunity to characterize pathways that drive disease risk and analyze associations between risk factors and health outcomes at the individual, household, community, and regional scale.

One potential limitation of the study is the existence of a subset of individuals (11.0%) within enrolled households that did not participate in the full set of data collection procedures employed in the complete study. The observation that a disproportionate percentage of the individuals that did not participate in the blood draw portion of the study were male (64.4%), for example, indicates that these missed individuals were not a random subset of the study population. However, participation rates for individuals within selected households were high (nearly 9 in 10 individuals completed a blood draw), and participation rates were highest among the groups (women and children) known to be most vulnerable to nutritional and disease risk. This suggests that, while future studies may benefit from additional emphasis on the recruitment and retention of adult men, our sample is unlikely to be significantly biased when assessing trends in disease in nutrition.

Further study of the sample material and data collected during this study is ongoing to characterize the highly variable contexts of health conditions in Madagascar and the vulnerability of rural communities therein. Indeed, preliminary data from this study indicates that more than 30 and 14% of the surveyed population had anemia or malaria infection, respectively. Future analyses will include: (1) the role of malaria and intestinal parasites in modulating nutritional status; (2) the role of proximity of forest or proximity to the sea in affecting micronutrient nutritional status; (3) the role of landscape diversity in affecting the diversity and abundance of larval mosquito vectors of disease and several others (see Supplementary Table 1). This study developed rich metadata associated with a suite of biological samples and nutritional and disease outcome data to allow examination of the underlying drivers of ill health across the diverse landscapes of Madagascar.

## Ethics Statement

All households were recruited and enrolled, and each individual consented or assented, following our IRB approved study (Protocol #16-0166, Committee on the Use of Human Subjects, Office of Human Research Administration at the Harvard T.H. Chan School of Public Health). The study was also reviewed and approved by the Malagasy Ministry of Health and the ethical review board at the Institut National de Santé Publique et Communautaire (INSPC) No 03/MSANP/SG/INSPC/DG/DFR. Informed consent was obtained from adults, verbal assent was obtained from children over 12 years of age, and permission was obtained from parents or guardians of younger children. Both the HSPH IRB and the INSPC review board waived the requirement for written informed consent for this study, and approved the study's consent procedures described previously because requiring signatures was deemed culturally inappropriate for the targeted populations.

## Author Contributions

CG, BR, and CM led overall study design and protocol development for the research. BR and HJR led the field research team in data collection. HJR supervised the surveyor research team and managed survey data collection. BR, AV, JR, AT, VR, AANAR, and RM performed the venous blood draws, blood sample processing, and clinical measurement data collection. MA, FL, RFFM, HPR, and ADR assisted design of the questionnaire and collected the questionnaire response data. NA, BR, and MT developed the study protocol on mosquito larval transects. NA and GE led the field data collection on mosquito larval transects and MT and RG identified mosquito specimens. BR, AA, AWe, AWi, CM, and DH led the infectious disease data analysis portion of the study. CG, JH, and CM sourced funding for the project. CG and BR drafted the manuscript. All authors read and approved the final manuscript. All authors contributed to the article and approved the submitted version.

## Conflict of Interest

The authors declare that the research was conducted in the absence of any commercial or financial relationships that could be construed as a potential conflict of interest.

## Abbreviations

ADRA: Adventist Development and Relief Agency
CRS: Catholic Relief Services
HSPH: Harvard T.H. Chan School of Public Health
MAHERY: Madagascar Health and Environmental Research
RDT: rapid diagnostic test
USAID: United States Agency for International Development
USD: United States Dollar.
